# Effect of zinc oxide or selenium nanoparticles on body weight, growth related genes and physiology in Baladi goats

**DOI:** 10.1038/s41598-025-23607-6

**Published:** 2025-11-04

**Authors:** Ibrahim M. Farag, Wael M. Aboulthana, Shimaa M. Ali, Mohamed I. El Sabry, Mahmoud Y. Mohamed, Mahmoud E. Abd El-Aziz, Mariam G. Eshak, Wagdy K. B. Khalil, Hayam Mansour

**Affiliations:** 1 Department of Cell Biology, Biotechnology Research Institute, National Research Centre, 33 El Bohouth St, P.O. 12622, Dokki, Giza Egypt; 2 Biochemistry Department, Biotechnology Research Institute, National Research Centre, 33 El Bohouth St, P.O. 12622, Dokki, Giza Egypt; 3 Animal Production Research Institute (APRI), Agricultural Research Center (ARC), Dokki, 12611 Giza Egypt; 4 Animal Production Department, Faculty of Agriculture, Cairo University, 6 El-Gamma St, Giza, 12613 Giza Egypt; 5 Polymers and Pigments Department, National Research Centre, 33 El Bohouth St, P.O. 12622, Dokki, Giza Egypt

**Keywords:** Zinc oxide nanoparticles, Selenium nanoparticles, Baladi goats, Body weight, Gene expression, Physiological parameters, Transcriptomics, Carbohydrates, Enzymes, Isoenzymes, Proteins, Proteomics, Animal biotechnology, Biochemical assays, Reverse transcription polymerase chain reaction, Nanoparticles, Proteomic analysis

## Abstract

Enhancing meat production is essential to meet the rising global demand for animal protein, improve food security, and support sustainable agriculture. This study evaluated the effects of zinc oxide (ZnO) and selenium (Se), each administered individually in conventional or nanoparticle (NP) forms on growth of Egyptian Baladi goats. Twenty-two pregnant Egyptian Baladi goats were divided into five groups, each receiving a different treatment via drinking water: 150 mg ZnO, 15 mg ZnO-NPs, 0.3 mg Se, or 0.03 mg Se-NPs. The control group received unsupplemented water. Treatments began 30 days before parturition and continued until weaning (90 days postpartum). Body weight, expression of GH, IGF-1, and leptin genes, along with physiological parameters, were evaluated. Goats receiving either ZnO-NPs or Se-NPs had significantly higher body weights at parturition and greater weight gain from birth to weaning than those in the conventional elements and control groups. Suckling kids from ZnO-NP or Se-NP-treated goats showed significantly higher birth and weaning weights, total body gain, and daily weight gain (*P* < 0.001), particularly in the ZnO-NP group. Gene expression analysis revealed upregulated GH and IGF-1 and leptin expression in ZnO-NP- and Se-NP-treated goats, with the highest levels observed in the Se-NPs group. Physiological analysis showed protein and esterase isoenzyme pattern changes in ZnO-treated goats, while Se caused no alterations. Neither ZnO nor Se affected catalase, peroxidase or α-amylase activity. These findings highlight the potential of ZnO-NPs and Se-NPs as effective and safe nutritional supplements for improving livestock productivity.

**Supplementary Information:**

The online version contains supplementary material available at 10.1038/s41598-025-23607-6.

## Introduction

Goats play a vital role in Egyptian agriculture, providing essential sources of meat and milk. The total goat population in Egypt is estimated at approximately 4.13 million heads, with the Baladi breed representing nearly 37% of this population. Baladi goats are widely distributed across the Nile Valley and Delta regions and are well-adapted to diverse environmental conditions. They play a significant role in smallholder farming systems, contributing to both meat and milk production in rural communities^1^. The economic viability of goat meat production is directly linked to body weight, which serves as a crucial metric for evaluating animal value and optimizing breeding programs^2^. Meat production traits result from a complex interplay of genetic and non-genetic factors, with nutrition playing a significant role^3^.

Recent advancements in nanotechnology have introduced the use of nanoparticles, such as ZnO-NPs and Se-NPs, as feed supplements to enhance production traits and promote growth rates in livestock. Although several studies have demonstrated the effectiveness of nanoparticles in improving growth performance in pigs^4^ and rabbits^5^, their impact in goat remains underexplored. In particular, no data are available on their influence on the expression of key growth-related genes. However, studies in goats and other animals have shown that nanoparticles can modulate the expression of genes related to various biological functions^6,7^.

Despite these potential benefits, metal nanoparticles may also pose risks by disrupting cellular homeostasis, causing DNA damage, and interfering with protein function^8^. Electrophoretic techniques serve as essential tools for detecting such alterations, allowing the assessment of DNA and protein variations and contributing to the understanding of their biological roles^9^.

Genetic factors are particularly significant as they govern various growth-related traits, including body weight, and shape phenotypic expression. Identifying these genetic factors is essential for effective herd management and breeding strategies. Key genes associated with growth regulation and development, and consequently body weights in farm animals include growth hormone (GH), insulin-like growth factor-1 (IGF-1), and leptin^10^.

The GH gene is a key regulator of growth and production traits in various livestock species, including goats^11,12^. Growth hormone encoded by GH gene stimulates body growth by promoting amino acid uptake and protein synthesis^13^. The insulin-like growth factor 1 hormone, encoded by the IGF-1 gene, is a structurally related protein that plays a critical role in cell proliferation, differentiation, metabolism regulation, embryogenesis, and overall growth. In goats, the IGF-1 gene has been shown to significantly influence growth across different breeds^14–16^. Owing to its pivotal role in regulating growth processes, IGF-1 has also been established as a valuable genetic marker for improving live weight and meat production in farm animals^10,13^. The third gene that play essential role in growth is leptin gene which encode leptin hormone. This gene and its hormone are responsible for regulating energy balance, feed intake, body weight, reproduction, and immune responses^17,18^.

Although the Egyptian Baladi goat is one of the most prominent purebred Egyptian breeds and is renowned for the quality and flavour of its meat, making it a valuable resource for livestock production, research into improving meat production in these goats remains scarce. The application of nanotechnology in livestock production has gained growing interest to enhance productivity in species such as goats, cattle, buffaloes, and sheep. In this context, the present study aims to evaluate the effect of ZnO-NPs or Se-NPs on body weight, growth-related gene expression (GH, IGF-1, and leptin), and physiological performance in Egyptian Baladi goats.

## Materials and methods

### Experimental animals

Twenty-two pregnant Baladi goats were used in this study, starting 30 days before their expected parturition, and were housed at the Animal Production Research Institute, Agricultural Research Center, Sids City, Beni Suef Governorate, Egypt. All experiments were performed in accordance with relevant guidelines and regulations and were approved by the Institutional Animal Care and Use Committee of the National Research Centre, Egypt (Ethical approval No. 13050410-2).

### Preparation of nanoparticles

ZnO-NPs were synthesized by refluxing 3.942 g of zinc acetate (Sigma Aldrich, St. Louis, USA) in 1 L of ethanol containing 1.44 g of NaOH (El-Gomhouria Co., Cairo) for 2 h at 70 °C. Following reflux, deionized water was added, and the mixture was centrifuged at 7000 rpm for 10 min. The resulting fine white powder was then calcined at 500 °C for 2 h to yield ZnO-NPs^19^. Se-NPs were prepared by dissolving 30 mg of sodium selenate (Na_2_SeO_3_·5 H_2_O, Sigma Aldrich, St. Louis, USA) in 90 ml of double-distilled water. To this solution, 10 ml of ascorbic acid solution (56.7 mM) was added dropwise while stirring vigorously. Polysorbate was added to a ratio of 0.01 ml to 2 ml of ascorbic acid. The successful synthesis of Se-NPs was confirmed by a visible color change from white to red^20^.

### Animal diet and sample collection

The goats were randomly divided into five groups (Table 1). Treatments were administered daily in 50 ml of drinking water and continued until weaning at 90 days postpartum.

**Table 1:** Experimental animal groups.

Group	Number of animals	Treatment
1	4	150 mg of conventional ZnO
2	5	15 mg of ZnO-NPs
3	4	0.3 mg of conventional Se
4	5	0.03 mg of Se-NPs
5	4	Unsupplemented drinking water

During the experimental period, which included parturition and lactation, body weights were recorded biweekly in the morning before feeding and drinking. Survival rates and feed intake were documented weekly, and body weight change was calculated by subtracting the initial weight (30 days prepartum) from the weight at weaning. At the end of the experiment, blood samples were collected from the jugular vein of all 22 goats in the morning before feeding to assess the expression of genes (GH, IGF-1, and leptin) associated with meat production and to perform physiological analysis.

### RNA extraction

RNA was extracted from blood samples of both control and treated animals, using TRIzol^®^ Reagent (Invitrogen, Germany). RNA purity (A260/A280 ratio: 1.8–2.1) was assessed using a NanoDrop spectrophotometer, and integrity were confirmed by agarose gel electrophoresis. Total RNA was treated with 1 U of RQ1 RNAse-free DNAse (Invitrogen, Germany) to digest DNA residues.

### Reverse transcription reaction

cDNA synthesis was performed using the RevertAid™ First Strand cDNA Synthesis Kit (MBI Fermentas, Germany) with 5 µg RNA in a 20 µL reaction. The reaction included 50 mM MgCl2, 5x reverse transcription buffer, 10 mM of each dNTP, 50 µM oligo-dT primer, 20 U ribonuclease inhibitor, and 50 U M-MuLV reverse transcriptase. The reaction was conducted in a thermocycler (Biometra GmbH, Göttingen, Germany) at 25 °C for 10 min, followed by 42 °C for 1 h.

### Quantitative real time PCR (qRT-PCR)

Gene expression levels were quantified using the StepOne™ Real-Time PCR System (Applied Biosystems, Thermo Fisher Scientific, Waltham, MA, USA). Each qRT-PCR reaction was prepared in a total volume of 25 µL, including SYBR^®^ Premix Ex Taq™ (TaKaRa, Biotech Co. Ltd.), 0.2 µM forward primer, 0.2 µM reverse primer, and 5 µL of cDNA template. The thermal cycling conditions were as follows: 95 °C for 3 min, followed by 40 cycles of 95 °C for 15 s, 55 °C for 30 s, and 72 °C for 30 s. Melting curve analysis was performed from 60 °C to 95 °C, with increments of 0.5 °C every 10 s. Relative gene expression levels were determined using the 2^−ΔΔCT^ method. The primer sequences for the target genes are listed in Table 2.

**Table 2:** Primer sequences used for qRT-PCR analysis of growth-related genes in goats.

Gene symbol	GenBank accession	Primer sequences (5′-3′)	References
GH	NM_001285586.1	F: GGCCCAGCAGAAATCAGACT R: CTTGAGCAGCGCGTCGTCAC	^21^
IGF-1	NM_001285697.1	F: TCGCATCTCTTCTATCTGGCCCTGT R: GCAGTACATCTCCAGCCTCCTCAGA	^22^
Leptin	XM_018046968.1	F: GCCTATGTGGGCATCCTTTA R: TGGAACAGGGAGGAAGACTG	^23^
GAPDH	XM_005680968.3	F: ATCAAGTGGGGTGATGCTGG R: GGCGTGGACAGTGGTCATAA	^24^

### Determination of total protein concentration

Plasma samples from each group were pooled by combining equal volumes from individual specimens into a single tube for analysis. The total protein concentration in the pooled samples was quantified using the Bradford method. To ensure consistent protein loading during electrophoretic assays, all samples were adjusted to equal concentrations by diluting them with loading dye.

### Native electrophoretic protein patterns

Native proteins were separated using Polyacrylamide Gel Electrophoresis (PAGE) and then stained with different dyes to visualize specific components. Coomassie Brilliant Blue (CBB) was used to visualize protein bands, appearing as blue bands. Sudan Black B (SBB) was employed to detect lipid components, which appeared as black bands. Alizarin Red (S) was utilized to identify calcium moieties, visualized as yellow bands, while Schiff’s reagent was used to detect carbohydrate moieties, appearing as pink bands.

### Native electrophoretic isoenzyme patterns

The electrophoretic patterns of Catalase (CAT) and Peroxidase (POX) were analyzed by incubating gels with hydrogen peroxide (H_2_O_2_) as a substrate. CAT isoforms were visualized as yellow bands following potassium iodide (KI) staining, while POX isoforms appeared as brown bands after benzidine staining. The α-amylase (α-Amy) pattern was assessed by incubating the native gel with a soluble starch solution, followed by iodine staining, which produced yellow α-Amy bands. Esterase (EST) isoenzymes were analyzed by incubating the gel in a conditioning buffer to optimize enzyme activity, followed by staining with a reaction mixture containing Fast Blue RR dye and α- or β-naphthyl acetate as substrates α-EST isoforms appeared as brown bands, while β-EST isoforms appeared as pink bands.

### Statistical analysis

Body weight data were expressed as mean ± SEM. A two-way repeated measures ANOVA was performed to evaluate the effects of treatment, time, and their interaction on body weight and growth parameters. Percentage values were arcsine-transformed before analysis and back-transformed for presentation. Data were analyzed using the Generalized Linear Model (GLM) procedure in SAS software version 9.2 (SAS Institute Inc., Cary, NC)^25^, and treatment means were compared using Tukey’s test at a significance level of *P* < 0.05.

Gene expression data were analyzed using the GLM procedure of SAS software version 9.2, followed by Scheffé’s test to determine significant differences among groups. Results are presented as mean ± SEM, with significance set at *P* < 0.05.

For physiological parameters, PAGE plate images were captured and analyzed using Quantity One software (Version 4.6.2) to determine relative mobility (Rf), quantity (Qty), and percentage contribution (B%) of protein bands. The similarity index (SI%) was calculated according to the method of Nei and Li^26^.

## Results

### Characterization of prepared zinc oxide and selenium nanoparticles

**Fig. 1:**
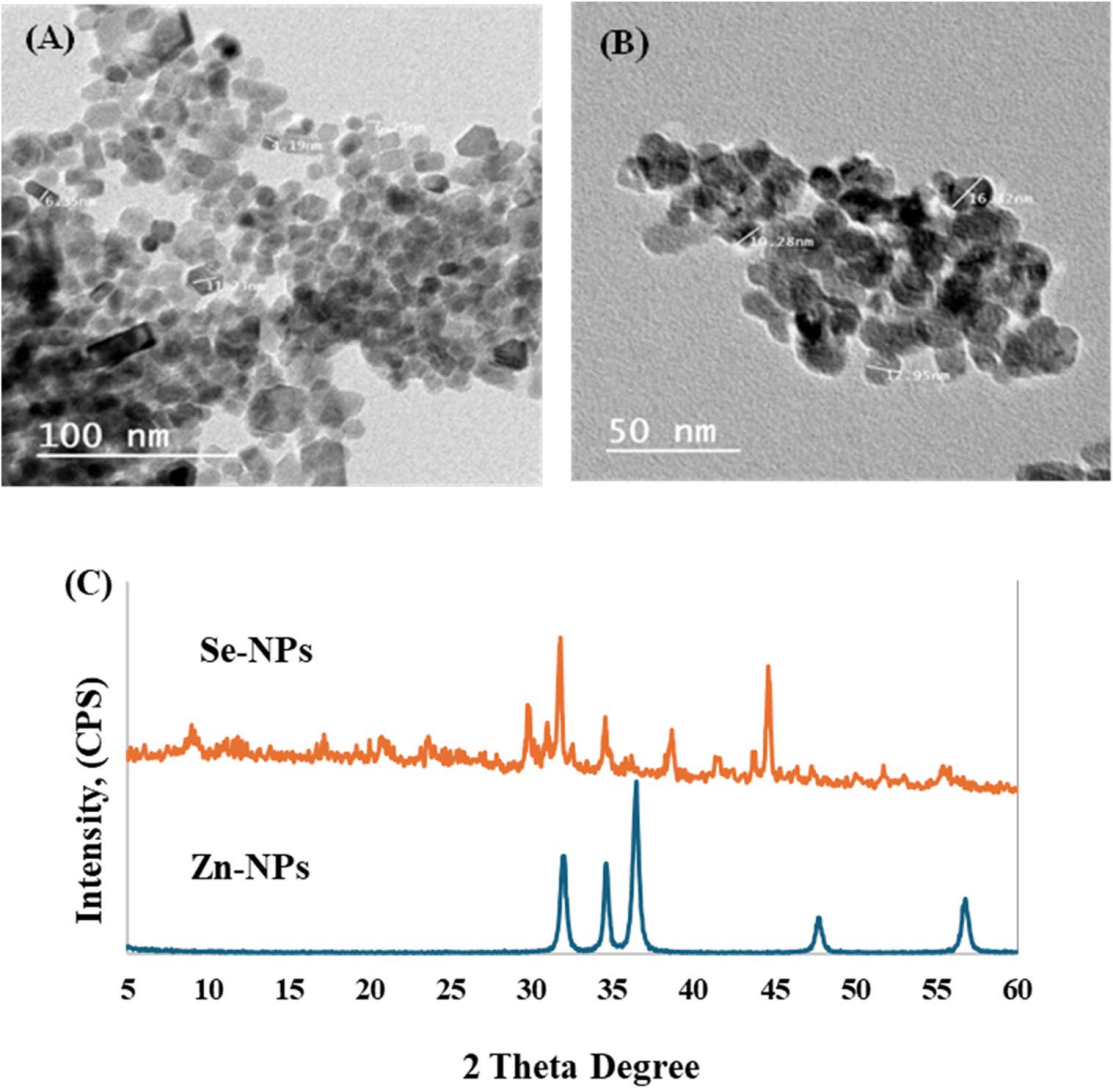
The TEM image of (**A**) ZnO-NPs and (**B**) Se-NPs as well as their XRD pattern (**C**).

Transmission Electron Microscopy (TEM) images of the synthesized ZnO-NPs and Se-NPs are shown in Fig. 1A, B, respectively. The ZnO-NPs had particle sizes of less than 100 nm (Fig. 1A), while the Se-NPs exhibited particles smaller than 50 nm (Fig. 1B). According to the Joint Committee on Powder Diffraction Standards (JCPDS) file format, the X-ray Diffraction (XRD) pattern of ZnO-NPs and Se-NPs is displayed in Fig. 1C. For ZnO-NPs, the configuration with planes at (100), (002), (102), and (110) are corresponding to peaks at 2θ = 32°, 34.4°, 36.4°, 47.7°, and 56.7°. For Se-NPs, the XRD diffraction peaks matched the pure hexagonal phase of selenium crystals with reflections at (100), (101), (111), (201), (003), and (210) that observed at 2θ = 23.9°, 30.0°, 45.7°, 52.0°, 55.9°, and 65.5°.

### Body weight evaluation of treated goats and their suckling kids

Mother Goats that received ZnO-NPs and Se-NPs from 30 days prepartum to the weaning of their offspring at 90 days postpartum exhibited significantly higher live body weights (LBW) and had significantly (*P* < 0.01) higher overall mean body weight than those of conventional element groups, while the control group showed the least weights. It is of interest to note that the LBW of ewes significantly (*P* < 0.05) showed a sharp reduction immediately after parturition and then a slight reduction up to 90 d postpartum in terms of LBW loss or as a percentage of change in LBW. The effect of interaction between treatments and experimental time was insignificant (*P* > 0.05) on the LBW of goats and was significant (*P* < 0.05) in BW loss and BW change, reflecting higher LBW of goats in NPs groups than in conventional elements and control groups at all experimental times (Table 3).

**Table 3:** Impact of zinc oxide or selenium nanoparticles on body weight and growth performance of Baladi mother goats.

Items	Day post -parturition	Experimental groups					± Standard error		
		Control	ZnO	Se	ZnO-NPs	Se-NPs	TR	D	TR_×_D
Live BW (kg)	− 30	34.00	34.25	34.25	34.20	34.60	0.277^***^	0.266^***^	0.620^ns^
	0^¥^	30.75	32.00	31.75	33.40	33.20			
	30	31.50	33.00	32.50	34.00	34.00			
	60	32.75	34.75	34.25	36.20	35.80			
	90	33.25	36.00	35.50	38.00	37.00			
	Overall	32.45^d^	34.00^bc^	33.65^c^	35.16^a^	34.92^ab^			
BW loss (kg)	− 30	–	–	–	–	–	0.160^***^	0.138^***^	0.321^*^
	0^¥^	− 3.25	− 2.25	− 2.50	− 0.80	− 1.40			
	30	0.75	1.00	0.75	0.60	0.80			
	60	1.25	1.75	1.75	2.20	1.80			
	90	0.50	1.25	1.25	1.80	1.20			
	Overall	− 0.19^c^	0.44^ab^	0.31^bc^	0.95^a^	0.60^ab^			
BW change (%)	− 30	–	–	–	–	–	0.466^***^	0.400^***^	0.932^*^
	0^¥^	− 9.56	− 6.57	− 7.30	− 2.34	− 4.05			
	30	2.44	3.13	2.36	1.80	2.41			
	60	3.97	5.30	5.38	6.47	5.29			
	90	1.53	3.60	3.65	4.97	3.35			
	Overall	− 0.41^b^	1.36^a^	1.02^ab^	2.73^a^	1.75^a^			

^¥^At parturition; TR, treatment; D, day; BW, body weigh; TR_×_D, interaction.

a, b, c and d, means with different superscripts, within each row are significantly different (*P* < 0.05).

The average LBW, monthly and daily weight gain, and % changes in kids’ BW at birth, 30, 60, and 90 days of age as affected by the treatments, kids’ age, and their interaction are presented in Table 4. Across all treatment groups, the average BW of neonatal kids increased relative to the control by 22.42% (ZnO), 14.45% (Se), 35.10% (ZnO-NPs), and 29.94% (Se-NPs). It is interesting to note that average BW, weekly and daily gain, and % change in BW of kids showed significant (*P* < 0.01) gradual increase by age progress, but only absolute and relative kids’ weight were affected significantly by the type of mother treatment. Both conventional and NP trace elements enhanced weight gain compared to the control, with the NP-supplemented groups achieving the most pronounced improvements.

**Table 4:** Impact of zinc oxide or selenium nanoparticles on body weight and growth performance of suckling kids.

Items	Day post -parturition	Experimental groups					± Standard error		
		Control	ZnO	Se	ZnO-NPs	Se-NPs	TR	D	TR_×_D
Live BW (kg)	0^¥^	1.79	2.24	2.01	2.52	2.41	0.120^***^	0.103^***^	0.241^***^
	30	5.77	6.93	6.85	7.63	7.24			
	60	8.67	10.75	10.04	11.83	11.44			
	90	10.90	13.28	12.12	14.66	14.13			
	Overall	6.78^e^	8.30^c^	7.76^d^	9.16^a^	8.81^b^			
Daily gain (g/d)	0^¥^	–	–	–	–	–	4.125^***^	3.065^***^	7.145^ns^
	30	132.67	156.33	161.33	170.33	161.00			
	60	96.67	127.33	106.33	140.00	140.00			
	90	74.33	84.33	69.33	94.33	89.67			
	Overall	101.22^d^	122.67^bc^	112.33^cd^	134.89^a^	130.22^ab^			
BW change (%)	0^¥^	–	–	–	–	–	7.366^ns^	5.473^***^	12.759^ns^
	30	222.35	209.38	240.80	202.78	200.41			
	60	50.26	55.12	46.57	55.05	58.01			
	90	25.72	23.53	20.72	23.92	23.51			
	Overall	99.44	96.01	102.69	93.92	93.98			
Survival rate (%)		100	100	100	100	100			

^¥^At parturition; TR, treatment; D, day; BW, body weigh; TR_×_D, interaction.

a, b, c and d, means with different superscripts, within each row are significantly different (*P* < 0.05).

### Expression analysis of body weight-related genes

**Fig. 2:**
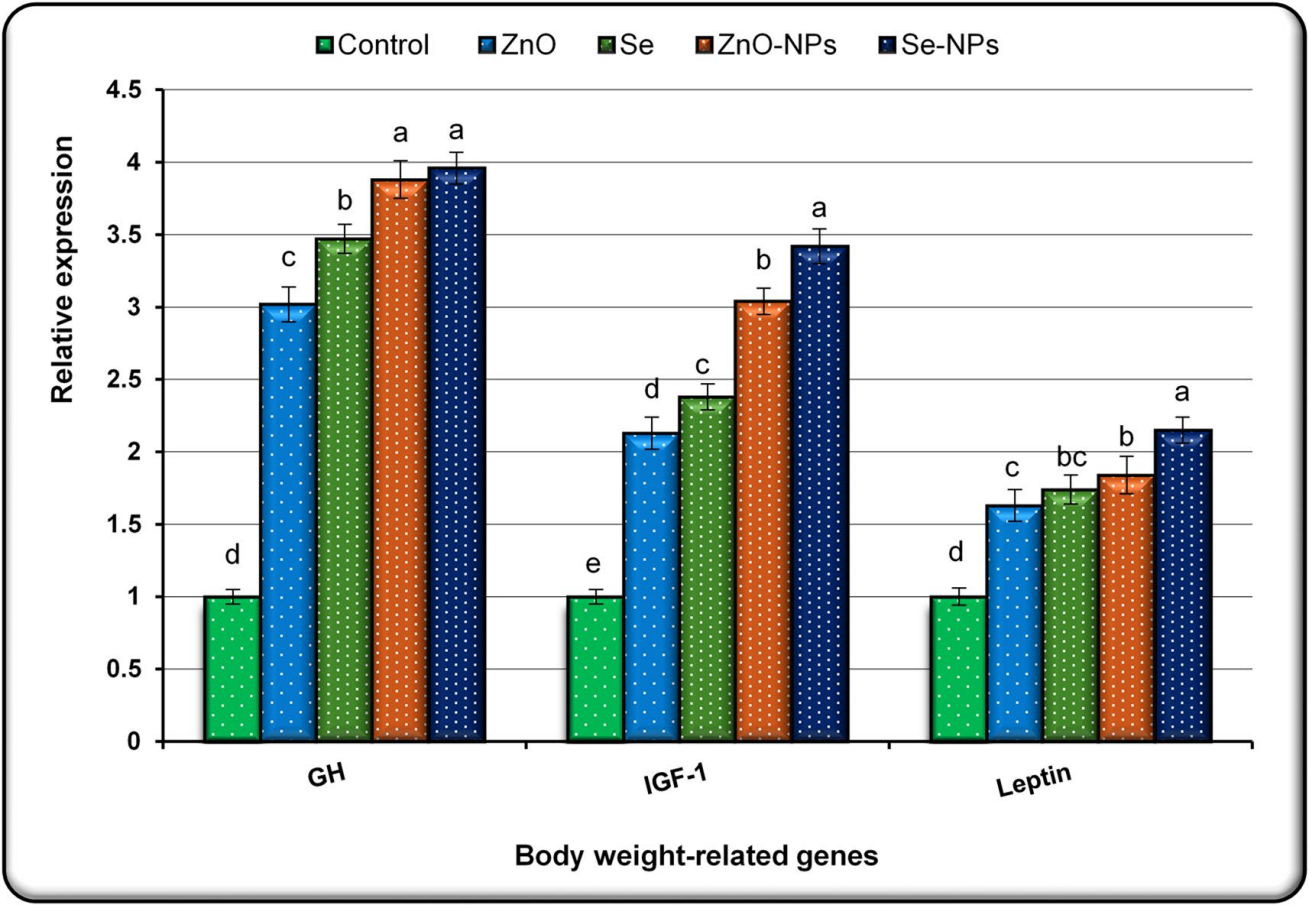
The expression alteration of GH, IGF-1 and Leptin genes in blood samples collected from mother goats in control and treated groups with ZnO, Se, ZnO-NPs and Se-NPs. Data are presented as mean ± SEM. ^a, b,c, d^: Mean values within treatments with unlike superscript letters were significantly different (*P* < 0.05).

The expression levels of body weight-related genes, including GH, IGF-1, and Leptin in mother goat groups treated with either the conventional or NP forms of ZnO and Se, are illustrated in Fig. 2. The data reveal that the control group exhibited significantly lower expression of all three genes compared to the treated groups. Among the conventional element treatments, animals received Se showed significant upregulation (*P* < 0.05) in the expression of GH and IGF-1 compared to those treated with ZnO. Although Leptin expression was slightly higher in the Se group than in the ZnO group, this difference was not statistically significant. When comparing with the NP treatments, Se-NPs demonstrated a pronounced enhancement in the expression levels of all three genes, with significant increases (*P* < 0.01) observed compared to both the control group and the conventional element-treated groups. Furthermore, animals treated with Se-NPs exhibited significantly higher (*P* < 0.05) expression of IGF-1 and Leptin expression levels compared to those treated with ZnO-NPs. However, the expression levels of GH were similar between the Se-NPs and ZnO-NPs groups, with no statistically significant differences noted.

### Physiological analysis and electrophoretic assay

#### Native protein and moiety patterns

**Fig. 3:**
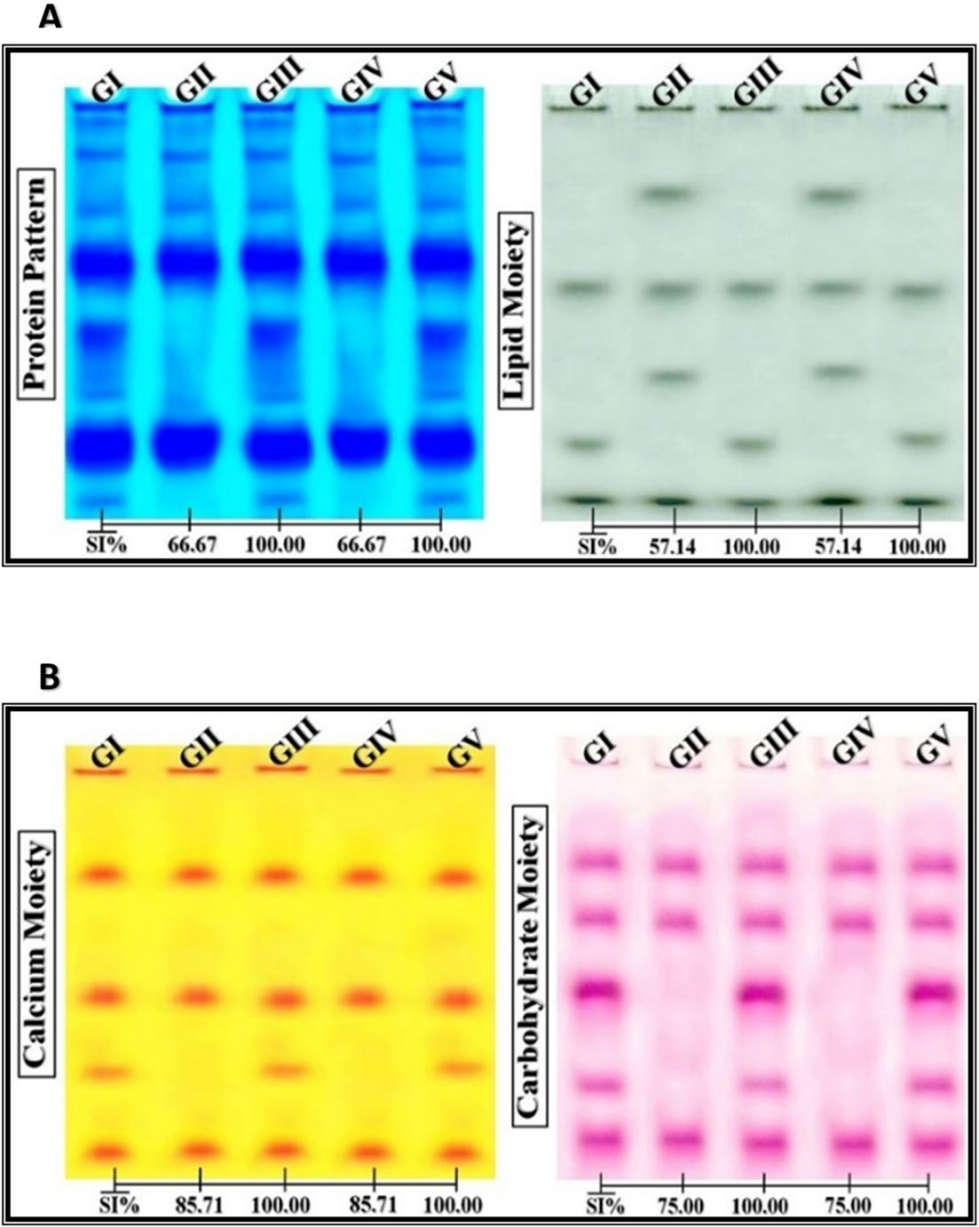
Native electrophoretic protein patterns showing the effect of ZnO-NPs and Se-NPs on the physiological properties of Egyptian Baladi goats, compared to conventional ZnO and Se. GI: Goats control, GII: Goats received ZnO, GIII: Goats received Se, GIV: Goats received ZnO-NPs, GV: Goats received Se-NPs. (**A**) shows the protein pattern and lipid moiety, (**B**) shows calcium moiety and Carbohydrate moiety.

No changes were observed in any protein or moiety patterns following treatment with Se or Se-NPs, with all profiles remained identical to the control group with 100% SI (Fig. 3A). In contrast, ZnO and ZnO-NP treatments induced several changes compared to the control group. Native protein patterns showed a reduction from eight to four bands, with an SI of 66.67%. The lipid profile was altered by the appearance of two new bands and loss of one, resulting in a 57.14% SI (Fig. 3A). In the calcium moiety, one band was lost (SI = 85.71%), and in the carbohydrate moiety, two bands disappeared (SI = 75%) (Fig. 3B).

### Native electrophoretic isoenzyme patterns

**Fig. 4:**
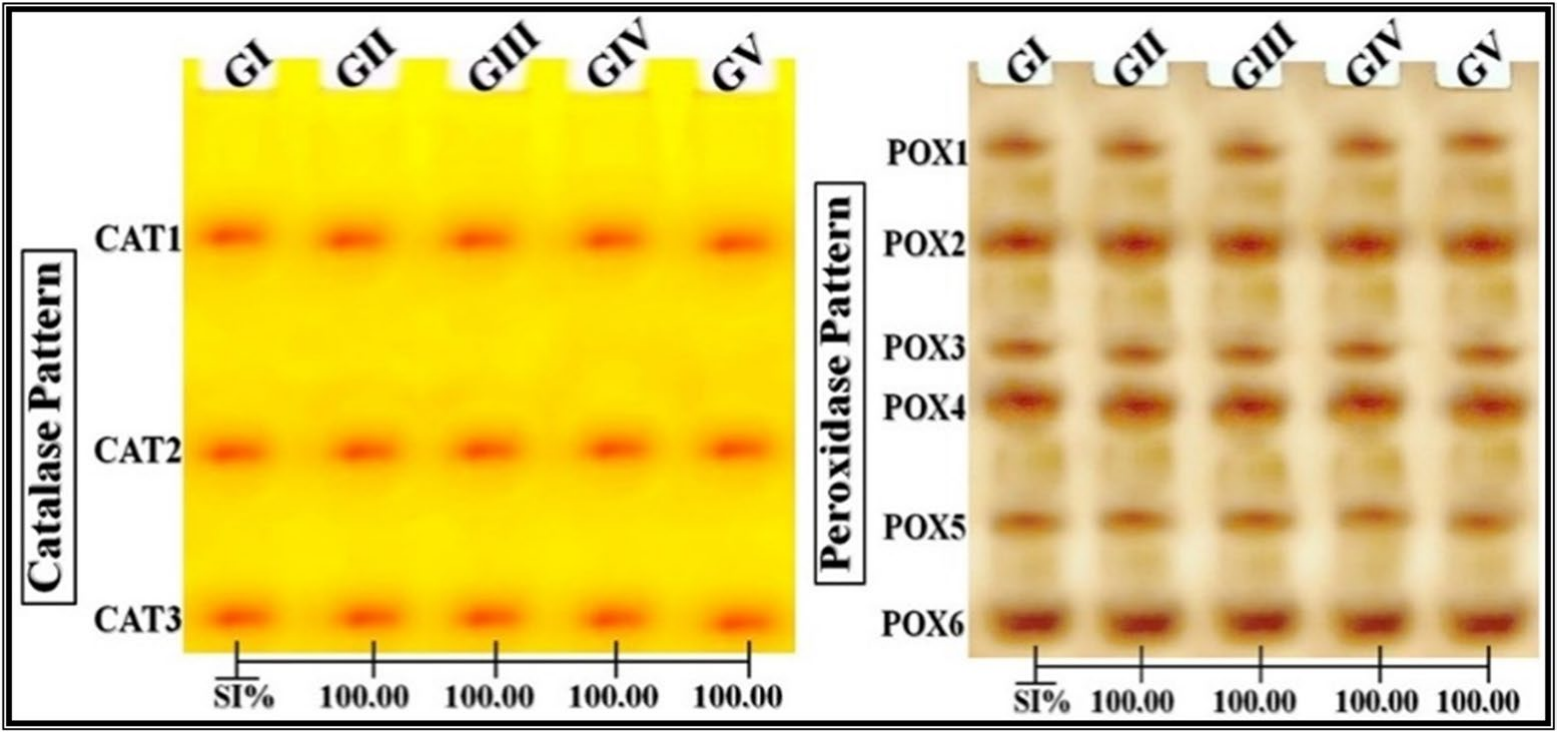
Native electrophoretic isoenzymes patterns showing the effect of ZnO-NPs and Se-NPs on the physiological state of catalase (CAT) and peroxidase (POX) patterns in Egyptian Baladi goats, compared to conventional ZnO and Se. GI: Goats control, GII: Goats received ZnO, GIII: Goats received Se, GIV: Goats received ZnO-NPs, GV: Goats received Se-NPs.

**Fig. 5:**
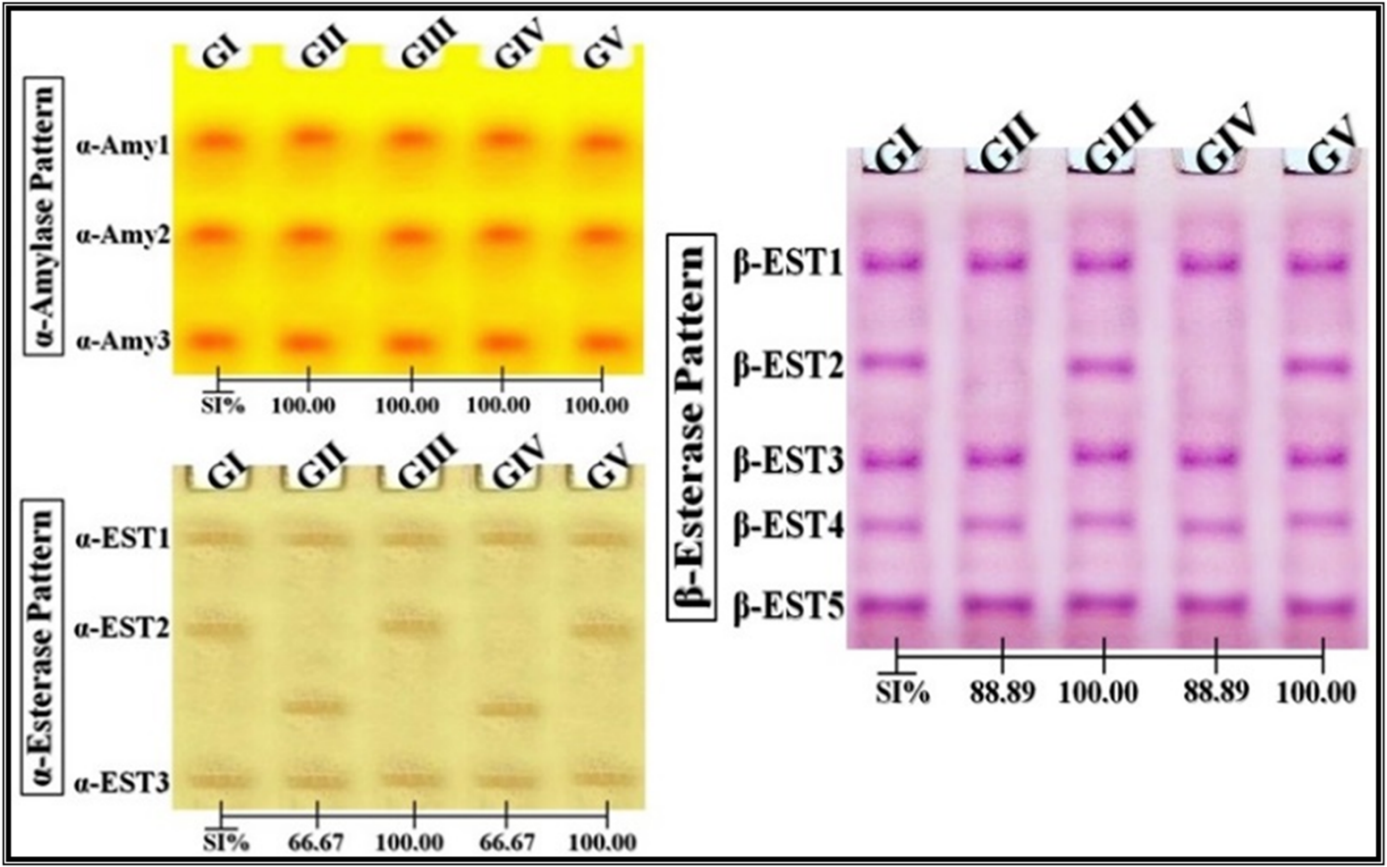
Native electrophoretic isoenzymes patterns showing the effect of ZnO-NPs and Se-NPs on the physiological state of α-amylase (α-Amy), α-esterase (α-EST) and β-esterase (β-EST) in Egyptian Baladi goats, compared to conventional ZnO and Se. GI: Goats control, GII: Goats received ZnO, GIII: Goats received Se, GIV: Goats received ZnO-NPs, GV: Goats received Se-NPs.

CAT, POX (Fig. 4), and α-Amy (Fig. 5) isoenzyme patterns were unaffected by all treatments, maintaining 100% similarity to the control. Alterations were observed only in ZnO-treated groups for EST isoenzymes: α-EST showed one lost and one new band compared to the control pattern (SI = 66.67%), while β-EST showed the loss of one band (SI = 88.89%). Se-treated groups showed no changes (Fig. 5).

Unprocessed and analyzed images of electrophoretic gels and isoenzyme patterns are provided in Supplementary File (Fig. S1-S9).

## Discussion

### The effect of NP elements on body weight of goats

The results in Table 3 show that mother goats receiving NP forms of ZnO or Se exhibited significantly higher body weights at parturition and throughout the postpartum period compared to those receiving conventional elements or no additives. Similarly, Table 4 demonstrates that kids from NP-treated groups had significantly higher birth and weaning weights, total body gain, and average daily gain compared to the conventional element-treated and control groups. Among all treatments, ZnO-NPs demonstrated the highest efficacy. These findings align with previous studies that reported enhanced growth performance following NP supplementation. The enhanced body weight observed in NP-treated goats may be attributed to improved nutrient bioavailability, more efficient feed utilization, and increased metabolic activity associated with nanoscale size that promotes absorption and cellular interactions^27,28^. ZnO-NPs have been shown to enhance growth and feed efficiency in several species. For instance, Lina et al.^29^ reported improved growth and carcass traits in broilers supplemented with 40 mg/kg ZnO-NPs. Similarly, Se-NPs supplementation has been associated with improved final body weight and average daily gain in goat bucks compared to control and sodium selenite-treated groups^30^. In rabbits, dietary supplementation with Se-NPs led to significant improvements in growth performance, weight gain, and carcass characteristics compared to natural Se sources^31^. The superior performance in trace element-treated groups may also relate to the essential roles of Zn and Se in enzymatic and hormonal processes. Compared to conventional forms, NPs offer higher biological availability due to their greater surface area and reactivity, resulting in enhanced catalytic efficiency^32,33^.

### Ther effect of NPs on gene expression and growth regulation

The gene expression results provide insights into the molecular mechanisms underlying the observed improvements in body weight and growth performance in NP-treated goats. Significant upregulation of GH, IGF-1, and leptin in these animals highlights the role of ZnO and Se, particularly in nanoparticle form, in modulating key growth-related pathways. GH and IGF-1 are critical for protein synthesis, cell proliferation, and muscle development, which likely contributed to the increased body weight and growth efficiency in treated groups^10,34^. The upregulation of leptin, a key hormone in regulating energy balance and fat metabolism, indicates enhanced metabolic efficiency and nutrient utilization in treated goats. Elevated leptin expression is typically linked to increased body fat and contributes to energy homeostasis by suppressing appetite and promoting metabolic activity^35^. Investigations in pigs and ewes have highlighted a significant connection between leptin levels and body fat accumulation^36,37^. Similarly, in cattle, leptin has been identified as a key factor influencing fat deposition, including backfat thickness and marbling quality^38^.

Although both conventional and NP forms showed positive effects, NPs demonstrated superior efficacy. ZnO-NPs and Se-NPs induced greater gene expression levels than their conventional counterparts, likely due to enhanced bioavailability and cellular uptake enabled by their nanoscale properties^32,33^. While limited data exist on the effects of ZnO-NPs and Se-NPs on gene expression in goats, findings from other species support our observations. In zebrafish, combined ZnO-NPs and Se-NPs supplementation increased GH and IGF-1 expression and improved growth outcomes^39^. In mice, ZnO-NPs upregulated genes involved in zinc metabolism, such as metallothionein, particularly at higher doses^40^. In goats, Abedin et al.^6^ reported that ZnO-NPs and Se-NPs supplementation in cryopreserved buck semen upregulated stress-related genes such as HSP70 and HSP90, further suggesting their protective and performance-enhancing roles.

Collectively, these findings support the use of ZnO-NPs and Se-NPs as effective modulators of growth-related gene expression. By enhancing the activity of GH, IGF-1, and leptin, these nanoparticles offer a promising strategy to improve meat yield and overall productivity in small ruminants.

### The effect of NPs on physiological parameters

To assess potential physiological risks, this study examined the effects of ZnO-NPs or Se-NPs on protein and isoenzyme patterns. Significant alterations were observed in the native protein profiles of goats treated with ZnO in both forms. These changes may be attributed to their ability to disrupt membrane fluidity and bind to protein thiol groups, particularly cysteine residues, leading to improper protein folding and inhibited enzyme function^41^. Additionally, ZnO-NPs release zinc ions that impair cellular energy production by downregulating key enzymes in glycolysis, the tricarboxylic cycle, and the electron transport chain^42^.

In terms of lipid-associated proteins, ZnO-NPs likely interact with plasma lipoproteins and acute-phase proteins, possibly adsorbing apolipoproteins via hydrophobic interactions through flexible hinge regions^43^. Moreover, ZnO-NPs can generate intracellular reactive oxygen species (ROS) in a size-dependent manner, inducing conformational and functional changes in proteins^44^. Calcium-binding proteins, typically involved in detoxification, also exhibited structural changes, potentially due to abnormal mineralization and ROS production triggered by ZnO-NPs^45^. Similarly, carbohydrate-linked protein patterns were disrupted, likely due to oxidative stress affecting glycosylation processes and protein stability^46^.

Enzymatic assays revealed alterations in the EST isoenzyme pattern in goats treated with ZnO in both forms, likely due to ROS-mediated polypeptide fragmentation and direct binding of ZnO-NPs to cysteine residues at the active site, forming enzyme-inhibitor complexes^47^. Other enzymes like CAT, POX and α-Amy were not affected by ZnO treatment. Conventional Se and Se-NPs did not affect native protein or isoenzyme profiles, suggesting a more favorable physiological safety profile.

In conclusion, oral administration of ZnO-NPs and Se-NPs improved body weight and enhanced the expression of growth-related genes in Baladi goats. The physiological changes observed in electrophoretic protein and isoenzyme patterns in the ZnO-NPs groups indicate potential metabolic effects, though no impairment in critical enzyme activities was detected. Se-NPs, however, did not result in such physiological alterations, suggesting that the impact of NP supplementation may vary based on the specific trace element used. NP supplementation may serve as a valuable approach to enhance bioavailability and biological activity. However, further research is warranted to explore the long-term effects and safety of these treatments in diverse animal models, as well as to optimize strategies for their practical application in livestock production systems.

## Supplementary Information

Below is the link to the electronic supplementary material.

Supplementary Material 1

## Data Availability

All data is provided within the manuscript or supplementary information files.
