# Supplementary material for: Effect of zinc oxide or selenium nanoparticles on body weight, growth related genes and physiology in Baladi goats
Source: Sci Rep. 2025 Nov 4;15:38477. doi: 10.1038/s41598-025-23607-6 (PMC12586602; doi:10.1038/s41598-025-23607-6)
Supplement: Supplementary file 1 — Supplementary Material 1 [file 41598_2025_23607_MOESM1_ESM.docx]

**Supplementary data**

**Effect of Zinc Oxide or Selenium Nanoparticles on Body Weight, Growth Related Genes and Physiology in Baladi Goats**

Ibrahim M. Farag^1.*^, Wael M. Aboulthana^2^, Shimaa M. Ali^3^, Mohamed I. El Sabry^4^, Mahmoud Y. Mohamed^3^, Mahmoud E. Abd El-Aziz^5^, Mariam G. Eshak^1^, Wagdy K.B. Khalil^1^, Hayam Mansour^1,*^

**The effect of NPs on physiological parameters**

This file contains the analyzed gel images corresponding to the electrophoretic and isoenzyme patterns presented in the main manuscript. Each figure is labeled according to its treatment group and staining method. These images are provided in compliance with the journal’s guidelines.


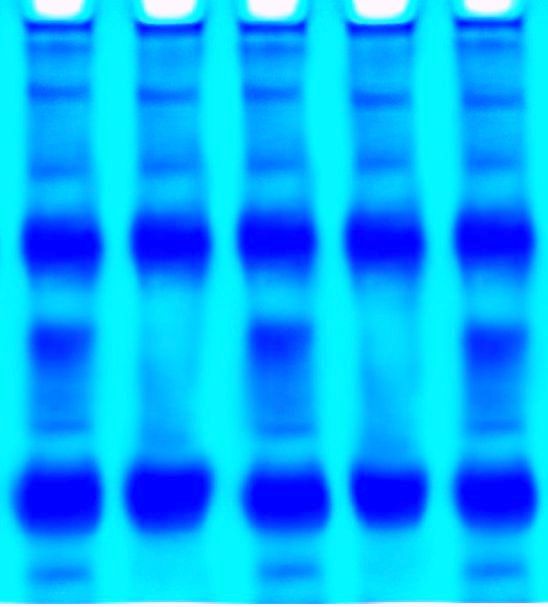


**GI GII GIII GIV GV**

**Supplementary Fig. S1a.** Native electrophoretic protein (Unprocessed Image) showing the effect of ZnO-NPs and Se-NPs on the physiological properties of Egyptian Baladi goats, compared to conventional ZnO and Se. GI: Goats control, GII: Goats received ZnO, GIII: Goats received Se, GIV: Goats received Zn-NPs, GV: Goats received Se-NPs.GIII: Goats received Se, GIV: Goats received Zn-NPs, GV: Goats received Se-NPs.

**
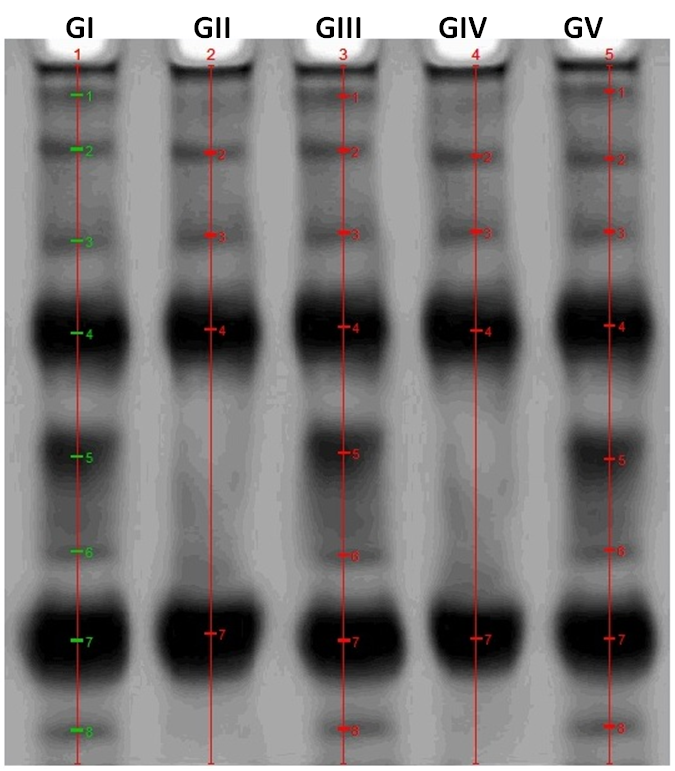
Supplementary Fig. S1b.** Native electrophoretic protein pattern (Unprocessed Analyzed Image) showing the effect of ZnO-NPs and Se-NPs on the physiological properties of Egyptian Baladi goats, compared to conventional ZnO and Se. GI: Goats control, GII: Goats received ZnO, GIII: Goats received Se, GIV: Goats received Zn-NPs, GV: Goats received Se-NPs.GIII: Goats received Se, GIV: Goats received Zn-NPs, GV: Goats received Se-NPs.

**
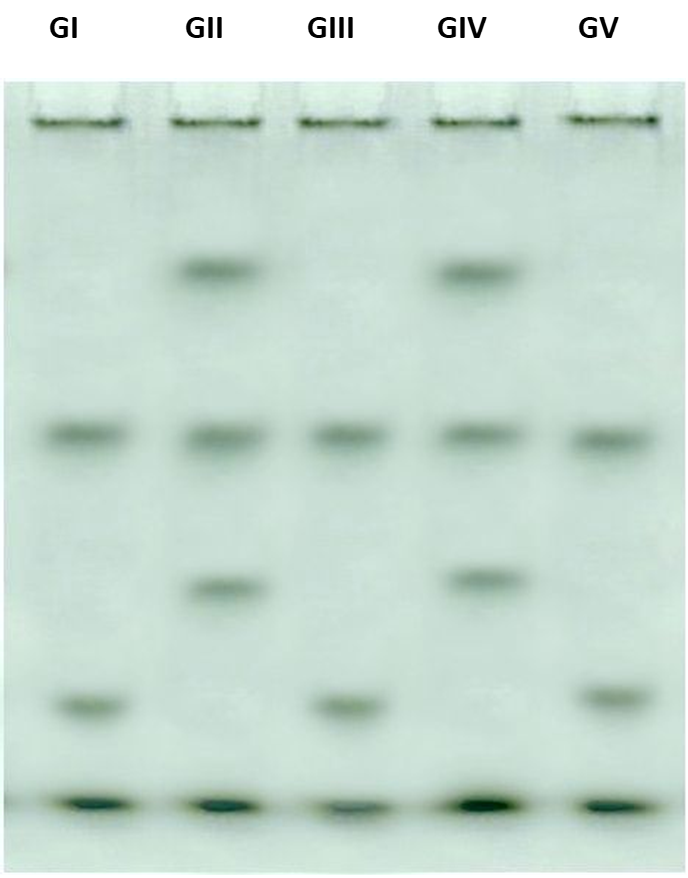
**

**Supplementary Fig. S2a.** Electrophoretic lipid moiety of native protein pattern (Unprocessed Image) showing the effect of ZnO-NPs and Se-NPs on the physiological properties of Egyptian Baladi goats, compared to conventional ZnO and Se. GI: Goats control, GII: Goats received ZnO, GIII: Goats received Se, GIV: Goats received Zn-NPs, GV: Goats received Se-NPs.


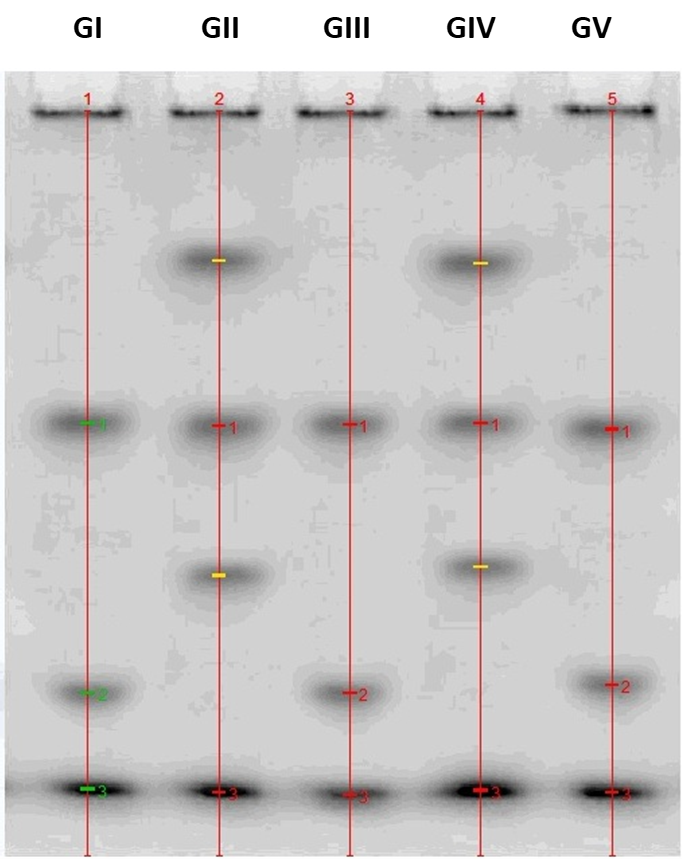


**Supplementary Fig. S2b.** Electrophoretic lipid moiety of native protein pattern (Unprocessed Analyzed Image) showing the effect of ZnO-NPs and Se-NPs on the physiological properties of Egyptian Baladi goats, compared to conventional ZnO and Se. GI: Goats control, GII: Goats received ZnO, GIII: Goats received Se, GIV: Goats received Zn-NPs, GV: Goats received Se-NPs.


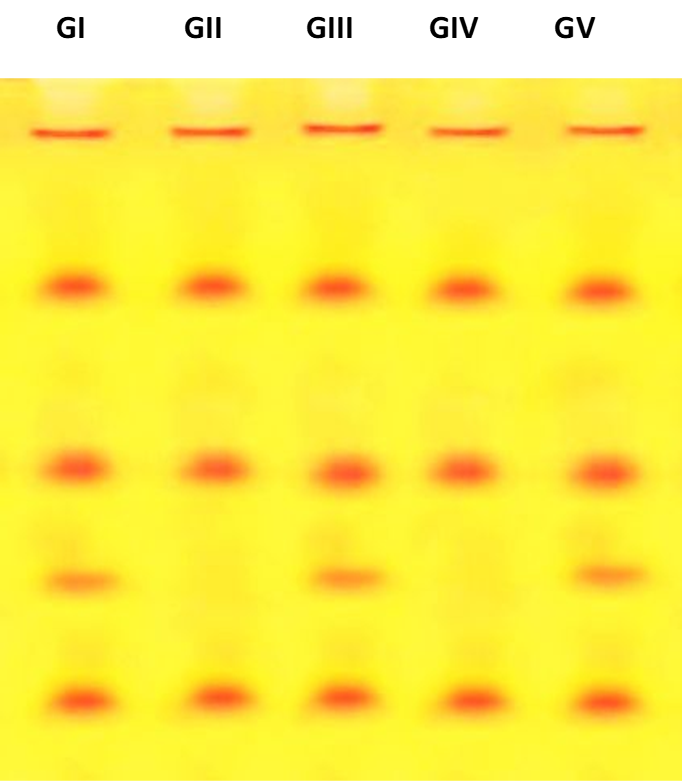


**Supplementary Fig. S3a.** Electrophoretic calcium moiety of native protein pattern (Unprocessed Image) showing the effect of ZnO-NPs and Se-NPs on the physiological properties of Egyptian Baladi goats, compared to conventional ZnO and Se. GI: Goats control, GII: Goats received ZnO, GIII: Goats received Se, GIV: Goats received Zn-NPs, GV: Goats received Se-NPs.


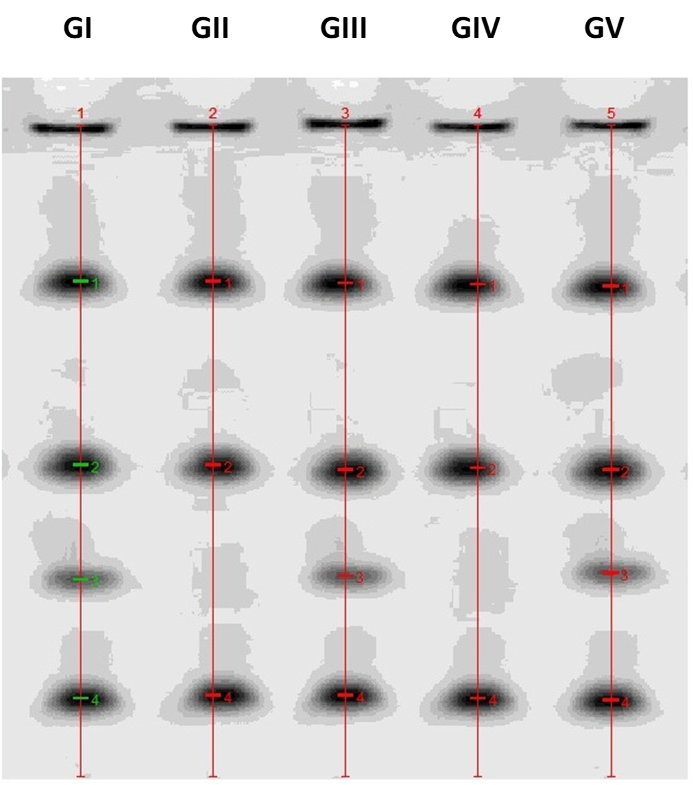


**Supplementary Fig. S3b.** Electrophoretic calcium moiety of native protein pattern (Unprocessed analyzed Image) showing the effect of ZnO-NPs and Se-NPs on the physiological properties of Egyptian Baladi goats, compared to conventional ZnO and Se. GI: Goats control, GII: Goats received ZnO, GIII: Goats received Se, GIV: Goats received Zn-NPs, GV: Goats received Se-NPs.


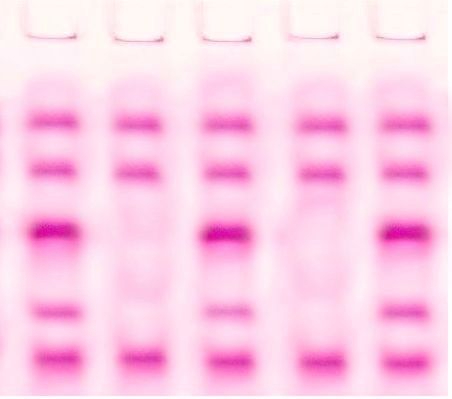


**GI GII GIII GIV GV**

**Supplementary Fig. S4a.** Electrophoretic carbohydrate moiety of native protein pattern (Unprocessed Image) showing the effect of ZnO-NPs and Se-NPs on the physiological properties of Egyptian Baladi goats, compared to conventional ZnO and Se. GI: Goats control, GII: Goats received ZnO, GIII: Goats received Se, GIV: Goats received Zn-NPs, GV: Goats received Se-NPs.


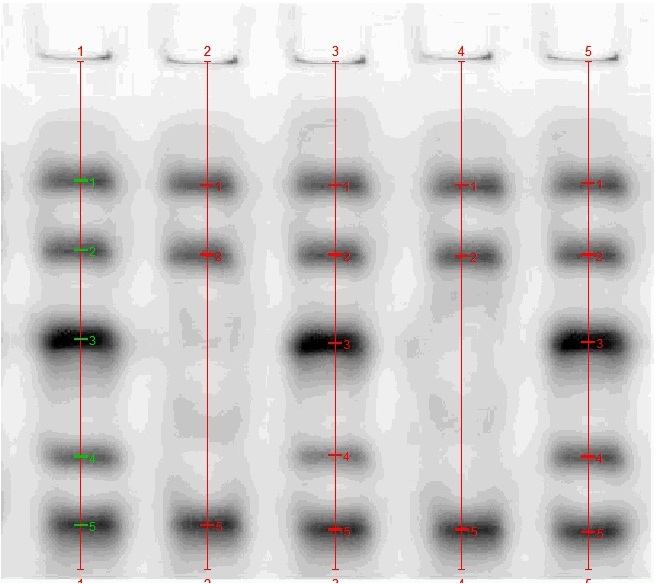


**GI GII GIII GIV GV**

**Supplementary Fig. S4b.** Electrophoretic carbohydrate moiety of native protein pattern (Unprocessed Analyzed Image) showing the effect of ZnO-NPs and Se-NPs on the physiological properties of Egyptian Baladi goats, compared to conventional ZnO and Se. GI: Goats control, GII: Goats received ZnO, GIII: Goats received Se, GIV: Goats received Zn-NPs, GV: Goats received Se-NPs.


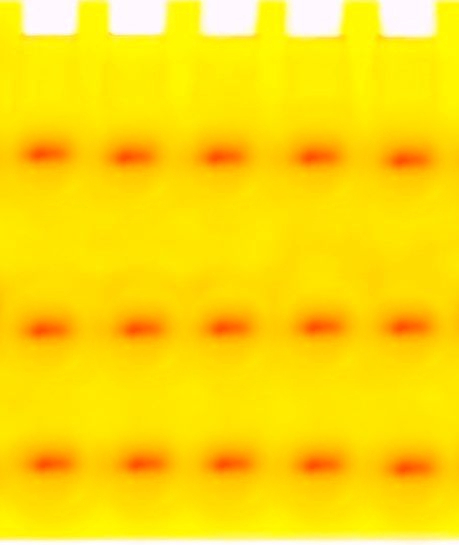


**GI GII GIII GIV GV**

**Supplementary Fig. S5a.** Electrophoretic catalase (CAT) isoenzyme pattern (Unprocessed Image) showing the effect of ZnO-NPs and Se-NPs on the physiological properties of Egyptian Baladi goats, compared to conventional ZnO and Se. GI: Goats control, GII: Goats received ZnO, GIII: Goats received Se, GIV: Goats received Zn-NPs, GV: Goats received Se-NPs.


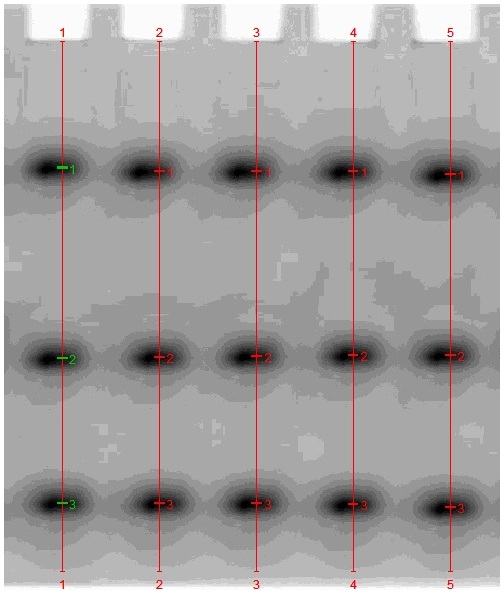


**GI GII GIII GIV GV**

**Supplementary Fig. S5b.** Electrophoretic catalase (CAT) isoenzyme pattern (Unprocessed Analyzed Image) showing the effect of ZnO-NPs and Se-NPs on the physiological properties of Egyptian Baladi goats, compared to conventional ZnO and Se. GI: Goats control, GII: Goats received ZnO, GIII: Goats received Se, GIV: Goats received Zn-NPs, GV: Goats received Se-NPs.


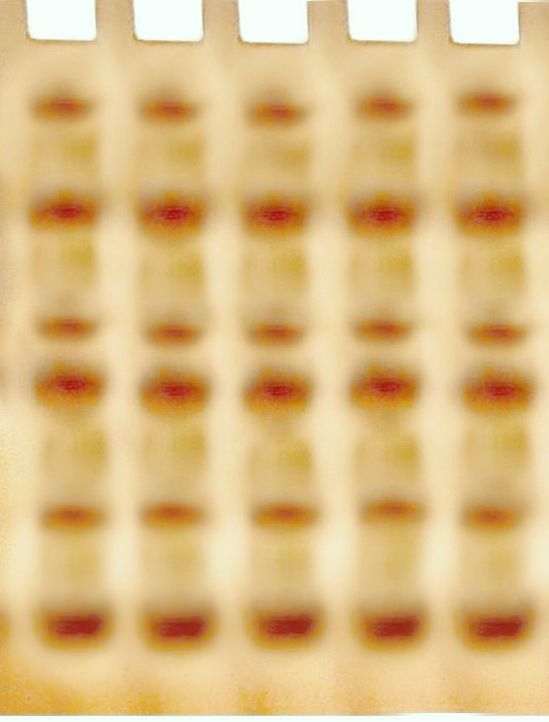


**GI GII GIII GIV GV**

**Supplementary Fig. S6a.** Electrophoretic peroxidase (POX) isoenzyme pattern (Unprocessed Image) showing the effect of ZnO-NPs and Se-NPs on the physiological properties of Egyptian Baladi goats, compared to conventional ZnO and Se. GI: Goats control, GII: Goats received ZnO, GIII: Goats received Se, GIV: Goats received Zn-NPs, GV: Goats received Se-NPs.

**GI GII GIII GIV GV**


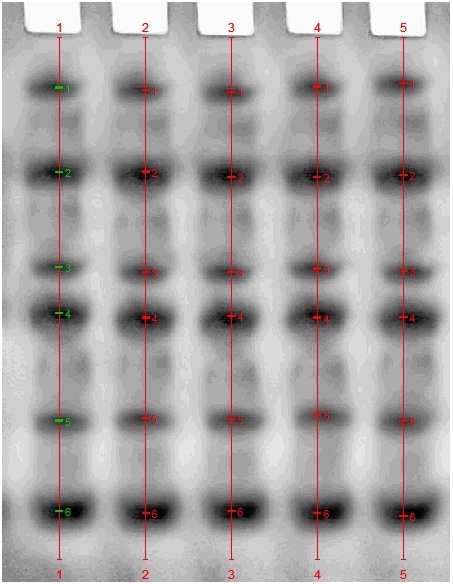
**Supplementary Fig. S6b.** Electrophoretic peroxidase (POX) isoenzyme pattern (Unprocessed Analyzed Image) showing the effect of ZnO-NPs and Se-NPs on the physiological properties of Egyptian Baladi goats, compared to conventional ZnO and Se. GI: Goats control, GII: Goats received ZnO, GIII: Goats received Se, GIV: Goats received Zn-NPs, GV: Goats received Se-NPs.


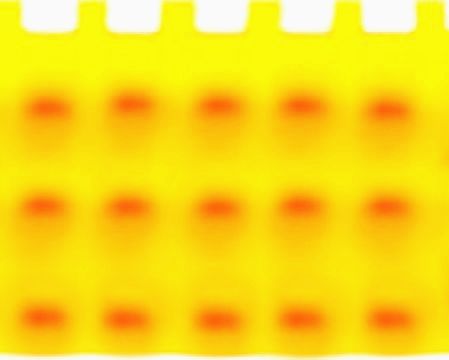


**GI GII GIII GIV GV**

**Supplementary Fig. S7a.** Electrophoretic α-amylase (α-Amy) isoenzyme pattern (Unprocessed Image) showing the effect of ZnO-NPs and Se-NPs on the physiological properties of Egyptian Baladi goats, compared to conventional ZnO and Se. GI: Goats control, GII: Goats received ZnO, GIII: Goats received Se, GIV: Goats received Zn-NPs, GV: Goats received Se-NPs.


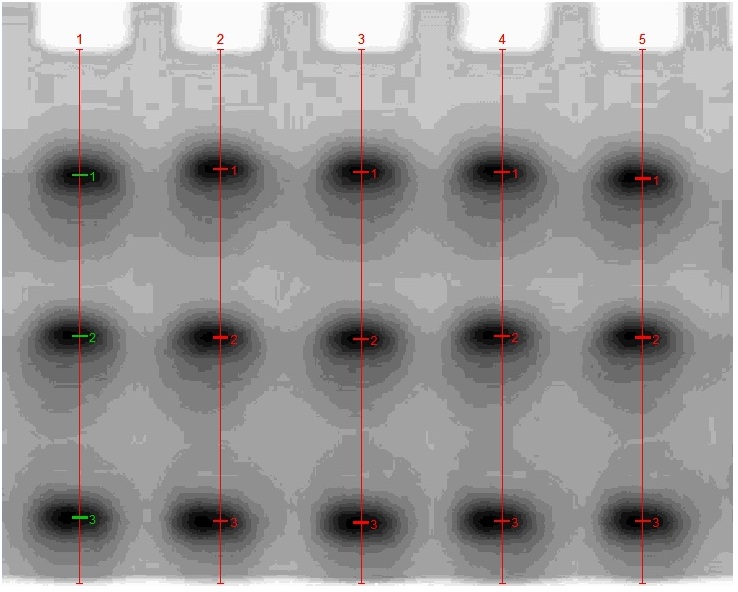


**GI GII GIII GIV GV**

**Supplementary Fig. S7b.** Electrophoretic α-amylase (α-Amy) isoenzyme pattern (Unprocessed Analyzed Image) showing the effect of ZnO-NPs and Se-NPs on the physiological properties of Egyptian Baladi goats, compared to conventional ZnO and Se. GI: Goats control, GII: Goats received ZnO, GIII: Goats received Se, GIV: Goats received Zn-NPs, GV: Goats received Se-NPs.


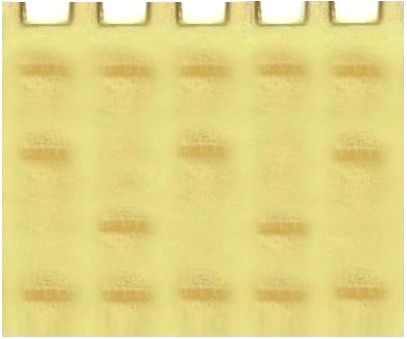


**GI GII GIII GIV GV**

**Supplementary Fig. S8a.** Electrophoretic α-esterase (α-EST) isoenzyme pattern (Unprocessed Image) showing the effect of ZnO-NPs and Se-NPs on the physiological properties of Egyptian Baladi goats, compared to conventional ZnO and Se. GI: Goats control, GII: Goats received ZnO, GIII: Goats received Se, GIV: Goats received Zn-NPs, GV: Goats received Se-NPs.


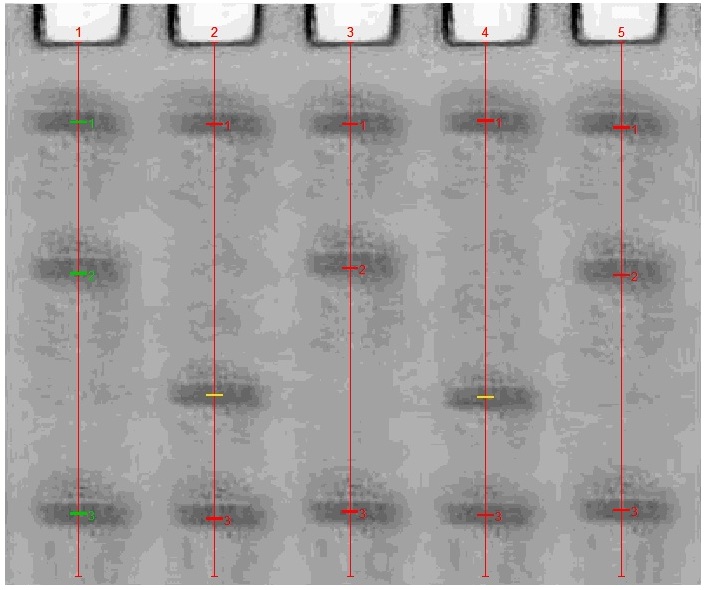


**GI GII GIII GIV GV**

**Supplementary Fig. S8b.** Electrophoretic α-esterase (α-EST) isoenzyme pattern (Unprocessed Analyzed Image) showing the effect of ZnO-NPs and Se-NPs on the physiological properties of Egyptian Baladi goats, compared to conventional ZnO and Se. GI: Goats control, GII: Goats received ZnO, GIII: Goats received Se, GIV: Goats received Zn-NPs, GV: Goats received Se-NPs.


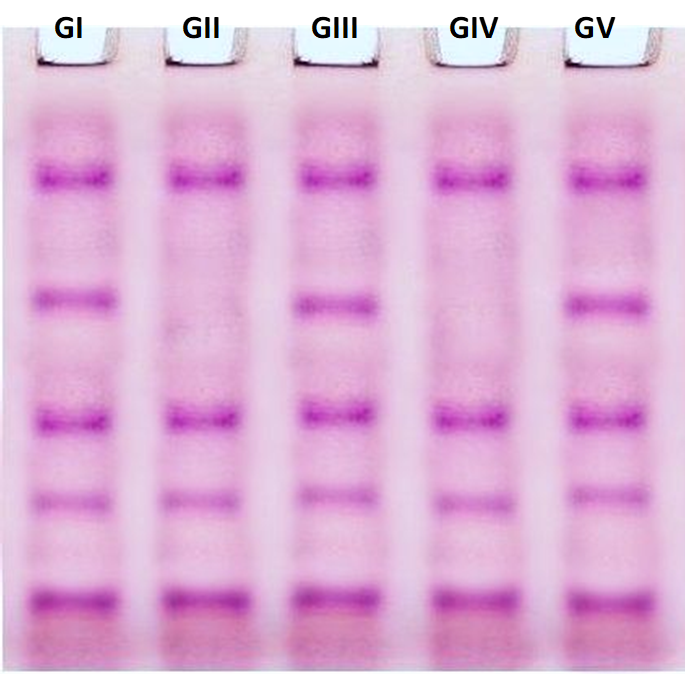


**Supplementary Fig. S9a.** Electrophoretic β-esterase (β-EST) isoenzyme pattern (Unprocessed Image) showing the effect of ZnO-NPs and Se-NPs on the physiological properties of Egyptian Baladi goats, compared to conventional ZnO and Se. GI: Goats control, GII: Goats received ZnO, GIII: Goats received Se, GIV: Goats received Zn-NPs, GV: Goats received Se-NPs.


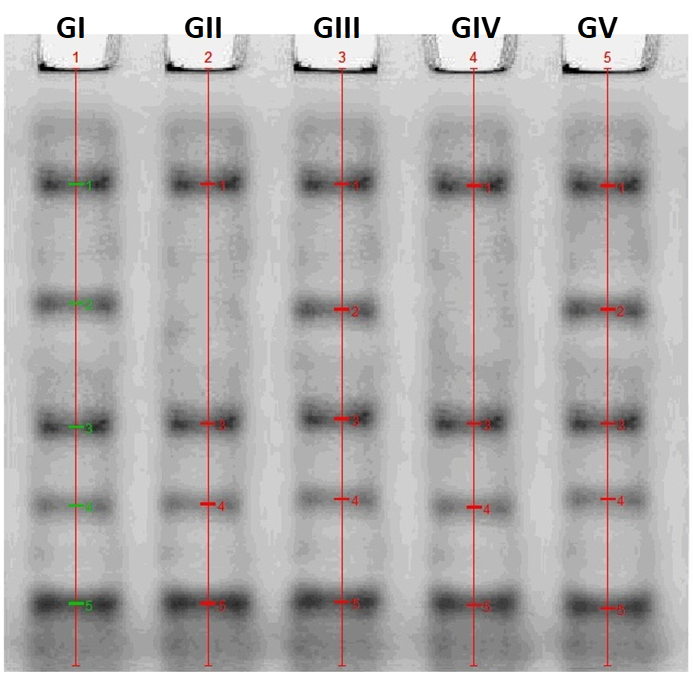


**Supplementary Fig. S9b.** Electrophoretic β-esterase (β-EST) isoenzyme pattern (Unprocessed Analyzed Image) showing the effect of ZnO-NPs and Se-NPs on the physiological properties of Egyptian Baladi goats, compared to conventional ZnO and Se. GI: Goats control, GII: Goats received ZnO, GIII: Goats received Se, GIV: Goats received Zn-NPs, GV: Goats received Se-NPs.
